# Prevalence of *pks* genotoxin among hospital-acquired *Klebsiella pneumoniae*

**DOI:** 10.3934/microbiol.2022007

**Published:** 2022-03-22

**Authors:** Amira H. El-Ashry, Shimaa R. Hendawy, Noha Mostafa Mahmoud

**Affiliations:** 1 Medical Microbiology & Immunology Department, Faculty of Medicine, Mansoura University, Mansoura, Egypt; 2 Clinical Pathology Department, Faculty of Medicine, Mansoura University, Mansoura, Egypt

**Keywords:** *Klebsiella pneumoniae*, *pks* genomic island, hypervirulent, colibactin, hypermucoviscous

## Abstract

The *pks* genotoxic *K. pneumoniae* has recently triggered a widespread alarm. DNA damage and higher virulence have been linked to colibactin, a genotoxin expressed by the *pks* genomic island. Little is known about its molecular epidemiology in clinical isolates from Egypt. Therefore, this study was conducted to determine the prevalence and the microbiological and clinical features of *pks* harboring hospital-acquired *K. pneumoniae* isolates from Egypt. Eighty-seven hospital-acquired *K. pneumoniae* isolates from various specimen types were screened for *pks* colibactin island markers *clbB*, *clbQ*, *clbA*, and *clbN* by PCR. The *pks*-positive hvKp isolates were classified to one of the capsular types K1 and K2 using multiplex-PCR targeting *K*-serotype *wzi* and *rmpA* genes. The prevalence of *pks^+^* strains was 27.6% (24/87). K1 capsular type, phenotypic, and genotypic hypervirulent isolates were significantly higher among *pks^+^* strains than *pks^−^* strains (*P* < 0.001), while *pks^+^ K. pneumoniae* strains were found to be significantly less resistant to 8 of the antibiotic compounds tested than *pks^−^* strains. Carriage of K1 capsular type and mucoviscosity-associated *rmp A* gene and diabetes mellitus were identified to remain independent risk factors having a substantial association to *pks*-positivity by multivariate regression analysis. In conclusion, Hospital-acquired *K. pneumoniae* isolates in Egypt had an increased prevalence of the *pks* colibactin genotoxin. The significant occurrence of hypervirulent determinants in *pks^+^ K. pneumoniae* highlighted the genotoxin's possible pathogenicity combined with its distribution in several specimen types, which necessitates clinical attention and epidemic tracking.

## 1. Introduction

*Klebsiella pneumoniae* (*K. pneumoniae*) is a potential nosocomial superbug that is generating clinical concern [1]. Hypervirulent *K. pneumoniae* (hvKp), which includes K1 and K2 strains, frequently shows hypermucoviscous phenotypes and carries a variety of hypervirulence genes [2],[3]. Worryingly, hvKp has spread worldwide, causing serious metastatic infection, particularly in immunocompetent persons, besides the emergence of multidrug-resistant (MDR) hypervirulent strains, all creating a major challenge in the clinical field [4].

The *pks* genomic island, detected in the *Escherichia coli* meningitis strain IHE3034 in 2006, transcodes for a hybrid of nonribosomal peptide synthetase-polyketide synthase enzymes of colibactin, a genotoxin that has been linked to DNA double-strand breaks, chromosome abnormalities, cell cycle arrest, and immune cell death. The *pks* island contains 19 colibactin synthesis genes (*clbA*-*clbR*); only the presence of all *pks* genes is required for complete colibactin production [5]. Bacteremia, meningitis, and colorectal cancer have all been correlated to the carriage of the *pks* gene in *E. coli* [6],[7]. Other Enterobacteriaceae members, such as *Citrobacter koseri*, *Klebsiella pneumoniae*, and *Enterobacter aerogenes*, have also been reported to contain the colibactin island [8]. Lately, the *pks* genes were also detected on sequencing of emerging hvKp and were linked to increased virulence [9].

While colibactin is considered a virulent factor in *K. pneumoniae*, little is documented about its molecular epidemiology in clinical isolates from Egypt. Therefore, this research aimed to determine the prevalence of *pks* island and investigate the microbiological and clinical features of *pks* harboring hospital-acquired *K. pneumoniae* isolates from Egypt.

## 2. Materials and methods

### 2.1. Bacterial isolates

Eighty-seven isolates of *K. pneumoniae* recovered by the Medical Microbiology and Infection Control Unit (MDICU), Mansoura University, Egypt, between May 2020 and August 2021 were attained for this observational cross-sectional study from clinical samples from 87 patients. Among them, 81.6% (71/87) were male, and the median age was 58 years ranging from (43–65 years). Our study included 58 isolates collected from urine and blood (29 each), 20 isolates from respiratory samples, and nine from other clinical samples. These isolates were identified, handled, and preserved using standard microbiological laboratory procedures [10]. A positive “string test” was used to define the hypermucoviscous traits. The string test was affirmative when a bacteriology inoculation loop can stretch bacterial colonies on an agar plate to form a viscous string > 5 mm in length [11].

### 2.2. Screening for pks genomic island, capsular types, and hvKp genes

The boiling method was employed to recover the genomic DNA template from a fresh culture of *K. pneumoniae* for PCR amplification [12]. PCR primers set for the four typical genes of the *pks* genomic island were used to develop two flanking (*clbB* and *clbQ*) and two internal (*clbA* and *clbN*) amplicons to document the existence of a full *pks* island in the isolates since they are clustered at the 3′ and 5′ ends of *pks* [13].

Based on multiplex-PCR amplification of *K*-serotype *wzi* and *rmpA* genes, the *pks*-positive hvKp isolates were classified to one of the capsular types K1 and K2 as documented previously [14]. Ethidium bromide staining and UV transillumination were used to visualize the PCR products on 1.5% agarose gel. The primers applied in the study and PCR programs are listed in table 1.

**Table 1.** Applied primers in the study.

Primer	DNA sequence (5′–3′)	PCR program	Amplicon size (bp)
*pks* genomic island			
*clbA*	F:CTAGATTATCCGTGGCGATTC R:CAGATACACAGATACCATTCA	10 min at 95 °C,30 cycles (45 sec at 94 °C, 45 sec at 54 °C, and 1 min at 72 °C); 10 min at 72 °C [12].	1311
*clbB*	F:GATTTGGATACTGGCGATAACCG R:CCATTTCCCGTTTGAGCACAC		579
*clbN*	F:GTTTTGCTCGCCAGATAGTCATTC R:CAGTTCGGGTATGTGTGGAAGG		733
*clbQ*	F:CTTGTATAGTTACACAACTATTTC R:TTATCCTGTTAGCTTTCGTTC		821

Capsular serotypes and Virulence gene			
K1	F: GGTGCTCTTTACATCATTGC R:GCAATGGCCATTTGCGTTAG	15 min at 95 °C,30 cycles (30 sec at 94 °C, 90 sec at 60 °C, and 90 sec at 72 °C); 10 min at 72 °C [13]	1283
K2	F:GACCCGATATTCATACTTGACAGAG R:CCTGAAGTAAAATCGTAAATAGATGGC		641
*rmpA*	F:ACTGGGCTACCTCTGCTTCA R:CTTGCATGAGCCATCTTTCA		516

### 2.3. Antimicrobial susceptibility pattern

*K. pneumoniae* isolates were tested for their antimicrobial sensitivity using disk diffusion testing, and results were interpreted as per the guidelines of the Clinical Laboratory Standards Institute (CLSI) for the breakpoints [15]. MDR-resistant isolates were those that were resistant to at least one antimicrobial agent in three or more of the classes [15]. *K. pneumoniae* ATCC 700603 was employed as quality control.

### 2.4. Data statistical analysis

SPSS software was used to analyze all data (version 25.0). The chi-square or Fisher's exact test were used to assess the significance of categorical variables. For continuous variables, the Mann-Whitney U test was used. Predictors of *pks*^+^ *K. pneumoniae* infection were analyzed using logistic regression. A multivariable logistic regression analysis was used to determine the association between predictor variables. Statistical significance was defined as a *p-*value < 0.05.

### 2.5. Ethics approval of research

The Institutional Review Board (IRB), Mansoura Faculty of Medicine, Egypt, code number: R/21.12.1539 approved the revision of patients' medical records and collection of bacterial isolates with the application of informed consent.

## 3. Results

Screening for *pks* genomic islands among our 87 *K. pneumoniae* isolates results in detecting colibactin structure markers represented in *clbA*, *clbB*, *clbN*, and *clbQ* amplified flanking and internal genes in 27.6% (24/87) isolates, which were classified as *pks^+^ K. pneumoniae* strains. Eleven of them were isolated from blood, eight from sputum, four from urine, and one from pus sample.

A total of 66.7% (58/87) of isolates were serotyped as K1 and K2. Capsular type K1 was prevalent in 45.9% (40/87) of isolates, while 20.6% (18/87) were classified as K2 capsular type. Twenty-nine isolates felt into other K1/K2 serotypes. According to data analysis, *pks^+^* strains had a considerably significantly high frequency of K1 capsular type than *pks^−^* strains (*p* < 0.001).

**Figure 1.**
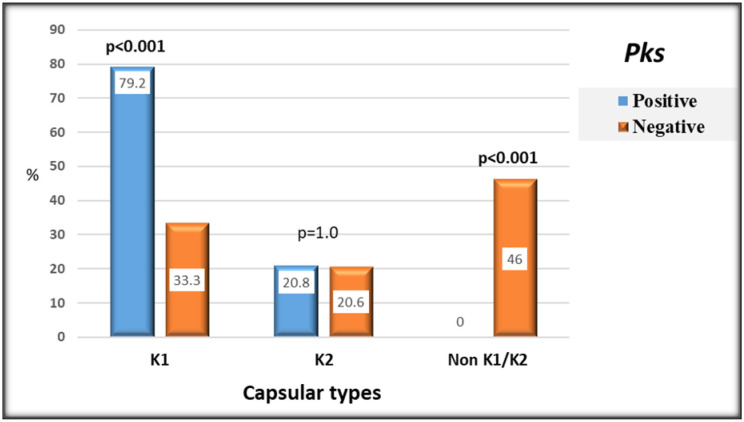
Capsular serotypes pattern of *pks*-positive and *pks*-negative *K. pneumoniae* isolates. *Note: Bold values denote statistical significance at the p < 0.05 level.

Hypermucoviscous isolates were significantly higher among *pks^+^ K. pneumoniae* compared to *pks^−^* isolates (*P* < 0.001) as detected phenotypically by positive string test in 54.2% (13/24) *pks^+^* isolates and genotypically by the presence of *rmpA* gene in 58.3% (14/24) *pks^+^* isolates. Data are shown in table 2.

**Figure 2.**
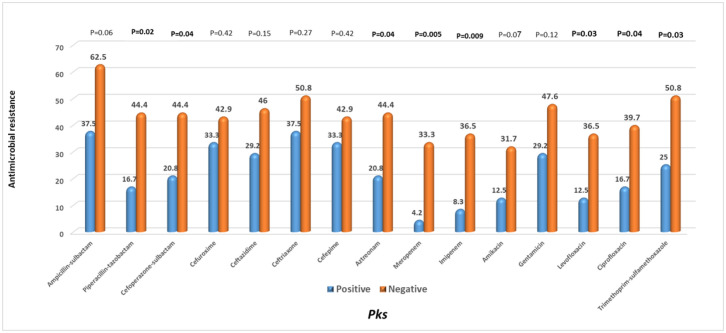
Antimicrobial resistance pattern of *pks*-positive and *pks*-negative *K. pneumoniae* isolates. *Note: Bold values denote statistical significance at the p < 0.05 level.

Regarding antimicrobial resistance, the *pks^+^ K. pneumoniae* isolates were less resistant to all antimicrobial agents tested versus *pks^−^* isolates. There was a statistically significant difference in susceptibility of *pks^+^* isolates to piperacillin-tazobactam, cefoperazone-sulbactam, aztreonam, meropenem, imipenem, levofloxacin, ciprofloxacin, and trimethoprim-sulfamethoxazole. Although there was a tendency toward more MDR *pks^−^* isolates, the difference was insignificant. Figure 2 provides an overview of the antimicrobial results.

**Table 2.** Factors associated with carriage of *pks* genomic island in hospital-acquired *K. pneumoniae* isolates.

	N (%) of *pks ^+^* isolates (n = 24)	N (%) of *pks ^−^*isolates (n = 63)	
Capsular types			<0.001*
K1	19 (79.2%)	21 (33.3%)	<0.001*
K2	5 (20.8%)	13 (20.6%)	1.00
Non K1/K2	0 (0%)	29 (46%)	<0.001*
Virulence factor			
Hypermucoviscous phenotype	13 (54.2%)	8 (12.7%)	<0.001*
Regulator of mucoid phenotype A (*rmp A*) gene	14 (58.3%)	11 (17.5%)	<0.001*
MDR	7 (29.2)	32 (50.8%)	0.07
Sample type			0.340
Blood	11 (45.8%)	18 (28.6%)	0.127
Respiratory	8 (33.3%)	21 (33.3%)	1.00
Urine	4 (16.7%)	16 (25.4%)	0.387
Others	1 (4.2%)	8 (12.7%)	0.227

*Note: A statistically significant *p* value < 0.05.

Concerning demographic and clinical data, patients above the age of 55 account for 70.1% of all infections, which may be due to increased susceptibility of old age to hospital-acquired infections. *pks ^+^ K. pneumoniae* infected patients were significantly younger than *pks ^−^* isolates infected patients (*P* = 0.028). There was no significant gender difference between the two groups. Diabetic patients were more vulnerable to *pks ^+^*isolates than *pks ^−^* isolates (*P* = 0.002) table 3.

**Table 3.** Demographic and Clinical characteristics of *pks*-positive and *pks*-negative *K pneumoniae*-infected patients.

	N (%) of *pks ^+^* isolates (n = 24)	N (%) of *pks ^−^*isolates (n = 63)	
Age, median, range	56 (50–65)	60 (43–61)	0.028*
Male	18 (75%)	53 (84.1%)	0.361
Underlying disorder			
Diabetes mellitus	17 (70.8%)	21 (33.3%)	0.002*
Hypertension	11 (45.8%)	17 (27%)	0.093
Pulmonary infection	10 (41.7%)	17 (27%)	0.186
Liver cirrhosis	5 (20.8%)	12 (19%)	1.00
Renal disorder	4 (16.7%)	9 (14.3)	0.747
Surgery within 30 days	2 (8.3%)	4 (6.3%)	0.666

*Note: A statistically significant *p*-value < 0.05

Carriage of K1 capsular type [Odds ratio (OR) 7.6, 95% confidence interval (95% CI) 2.49–23.194] and mucoviscosity-associated *rmp A* gene [OR 6.618, 95% CI 2.339–18.725], and diabetes mellitus [OR 4.857, 95% CI 1.744–13.528] were identified to remain independent risk factors having a substantial association to *pks*-positivity by multivariate regression analysis.

## 4. Discussion

Colibactin genotoxin, encoded by the *pks* island enzymes, has been linked to host DNA damage and higher virulence among *E. coli* and *K. pneumoniae,* resulting in severe infection sequelae [9],[17]. However, limited research data about the prevalence and microbiological variables related to *pks*-positivity in *K. pneumoniae* isolates.

In 24 (27.6%) out of the 87 clinically collected *K. pneumoniae* strains from patients with primary *K. pneumoniae* infections at a tertiary university hospital in Egypt, the *pks* colibactin island markers *clbB*, *clbQ*, *clbA*, and *clbN* were simultaneously detected. As a result, the prevalence rate was 27.6%, representing the first epidemiological study on a *pks* island hosting *K. pneumoniae* in Egypt.

Our findings backed up Lan et al. results, which documented a high *pks* prevalence in *K. pneumoniae* with 26.8% [18]. Positive frequencies of *pks* among *K. pneumoniae* recovered from different body sites were listed to be 25.6 % and 16.7 %, respectively, in two prior studies conducted in Taiwan [3],[19]. The *pks* gene was also highly prevalent in *E. coli* isolated from bacteremia patients, varying from 31.5 % to 58 % [20].

Hypervirulent variants are classified according to specific capsular serotypes, particularly K1 and K2 strains, which have been documented to be the most virulent of the 79 capsular types [21]. In this study, PCR screening-detected K1 type in 79.2% (19/24) of the colibactin-positive *K. pneumoniae*. The co-carriage of K1 type and colibactin genes was significantly correlated [OR 7.6, 95% CI 2.49–23.194, *P* < 0.001]. A close association of *pks* genes to K1 type was previously reported by Lai et al., who found that 66% (35/53) of the *pks ^+^* were K1 [19]. K1 or K2 capsular serotype-specific genes are located on the same virulence plasmid and other virulence encoding genes, which contributes to resistance to phagocytosis and intracellular killing by macrophages and neutrophils. This may partially explain the absence of non K1/K2 serotypes among *pks^+^* isolates [22],[23].

On the other side, the existence of genotoxic *K. pneumoniae* was unrelated to the type of infection since colibactin-positive isolates were insignificantly distributed among bacteremia (11/29; 37.9%), respiratory infection (8/29; 27.6%), urinary tract infection (4/20; 20%), and other cases (1/9; 11.1%).

Despite the widespread use *rmp A* gene for genotypic hvKp detection, the hypermucoviscous phenotype is still thought to be one of the most relevant virulence factors of hypervirulent strains [24]. More than half of our genotoxic *K. pneumoniae* clinical isolates were positive for hypermucoviscous phenotype and *rmp A* gene (54.2% and 58.3%, respectively); this was significantly higher than in the non-genotoxic group (*P* < 0.001). The growing evidence stated by preceding publications [3],[18], indicating that the colibactin genotype is linked to hypervirulent strains, is also supported by our results of significant correlation of *pks* genes and mucoviscosity-associated *rmp A* gene [OR 6.618, 95% CI 2.339–18.725]. More research is needed to determine if the *pks* genomic island contributes directly to virulence or acts as a signal tangled in pathogenesis.

The absence of a positive regulator required for full *rmpA* expression, or the presence of a negative regulator of Lon protease at the posttranscriptional level, could clarify the positivity for *rmpA* in the four phenotypically non-hypermucoviscous *K. pneumoniae* strains, which was previously detailed by Yu et al. [25].

The present study detected minimal antimicrobial resistance in *pks^+^* isolates, as Shi et al. [26] reported. According to statistical analysis, colibactin-positive isolates were significantly less resistant to 8 of the 15 antimicrobial compounds tested than colibactin-negative isolates. This was probably due to the high numbers of hypervirulent serovars and virulence genes found in *pks^+^* isolates, as virulence is typically associated with lower drug resistance. Alternatively, maybe partially attributable to the fact that all of these isolates were K1 and K2 capsular types, which are often less resistant to the antimicrobial agents [27],[28].

Multidrug-resistant hvKp strains that produce extended-spectrum lactamase (ESBL) or carbapenemase have been labeled a challenging scenario in clinical cases [29],[30]. MDR was detected in 29.2% of our colibactin-positive isolates relative to 50.8% in the negative group characterized from similar situations of low association of virulence with drug resistance. Even though the MDR frequency among *pks*-positive isolates is currently low, the evolution of MDR paired with genotoxicity is concerning. So antimicrobial resistance should be carefully monitored in these genotoxic isolates.

In line with other research [18], underlying disorders such as diabetes mellitus, as well as the K1 capsular type, were all significant risk factors for genotoxic *K pneumoniae* infections.

## 5. Conclusions

Hospital-acquired *K. pneumoniae* isolates in Egypt had an increased prevalence of the *pks* colibactin genotoxin. The presence of hypervirulent determinants in *pks^+^ K. pneumoniae* highlighted the genotoxin potential pathogenicity. Its distribution in several specimen types aroused concerns about genotoxic *K. pneumoniae* traits in various pathological conditions, which necessitates clinical attention and epidemic tracking.
